# Comparative Analysis of Extracellular Vesicles from Cytotoxic CD8^+^ αβ T Cells and γδ T Cells

**DOI:** 10.3390/cells13201745

**Published:** 2024-10-21

**Authors:** Lisa Griesel, Patrick Kaleja, Andreas Tholey, Marcus Lettau, Ottmar Janssen

**Affiliations:** 1 Molecular Immunology—Institute for Immunology, University Hospital Schleswig-Holstein, Campus Kiel, 24105 Kiel, Germany; 2 Systematic Proteomics & Bioanalytics—Institute for Experimental Medicine, University of Kiel, 24105 Kiel, Germany; p.kaleja@iem.uni-kiel.de (P.K.); a.tholey@iem.uni-kiel.de (A.T.); 3 Stem Cell Transplantation and Immunotherapy—Internal Medicine II, University Hospital Schleswig-Holstein, Campus Kiel, 24105 Kiel, Germany

**Keywords:** cytotoxic T cells, CD8^+^ T cells, αβ T cells, γδ T cells, cytotoxic granules, cytotoxic effector proteins, lysosome-related effector vesicles (LREVs), extracellular vesicles (EVs), exosomes, proteomics profiling

## Abstract

Background: Although belonging to different branches of the immune system, cytotoxic CD8^+^ αβ T cells and γδ T cells utilize common cytolytic effectors including FasL, granzymes, perforin and granulysin. The effector proteins are stored in different subsets of lysosome-related effector vesicles (LREVs) and released to the immunological synapse upon target cell encounter. Notably, in activated cells, LREVs and potentially other vesicles are continuously produced and released as extracellular vesicles (EVs). Presumably, EVs serve as mediators of intercellular communication in the local microenvironment or at distant sites. Methods: EVs of activated and expanded cytotoxic CD8^+^ αβ T cells or γδ T cells were enriched from culture supernatants by differential and ultracentrifugation and characterized by nanoparticle tracking analyses and Western blotting. For a comparative proteomic profiling, EV preparations from both cell types were isobaric labeled with tandem mass tags (TMT10plex) and subjected to mass spectrometry analysis. Results: 686 proteins were quantified in EV preparations of cytotoxic CD8^+^ αβ T cells and γδ T cells. Both populations shared a major set of similarly abundant proteins, while much fewer proteins presented higher abundance levels in either CD8^+^ αβ T cells or γδ T cells. To our knowledge, we provide the first comparative analysis of EVs from cytotoxic CD8^+^ αβ T cells and γδ T cells.

## 1. Introduction

Cytotoxic CD8^+^ TCRαβ^+^ T cells and TCRγδ^+^ T cells eliminate virus-infected or transformed cells by local exposure and release of cytotoxic effector proteins. Lysosome-related effector vesicles (LREVs) [1], previously also termed secretory lysosomes (SLs) [2], were identified as intracellular storage compartments for FasLigand (FasL), granzymes, perforin and granulysin [3,4,5]. Upon activation or target cell encounter, such vesicles are transported to the cytotoxic immunological synapse which forms between the effector and the target cell [6,7]. This structure ensures a local release and exposure of effector proteins to induce target cell death without collateral damage. We and others previously identified at least two subtypes of LREVs in healthy T and NK cell subsets [8,9,10,11,12]. Importantly, these morphologically distinct vesicles are differentially mobilized upon activation [10,13]. Light vesicles carrying FasL and the 15 kDa form of granulysin degranulate in a non-classical PKC-dependent but calcium-independent fashion. In contrast, dense granules that contain granzyme B or perforin and the mature 9 kDa granulysin are released by classical degranulation and thus require calcium-dependent signals [13,14]. As a putative mechanism for differential mobilization, we reported that light and dense granules are associated with different actin and myosin structures for their intracellular movement [9,13].

In recent years, an increasing number of reports have emphasized that T and NK cells do not only store effector molecules and secrete them locally into the IS, but they may also release them constitutively or after activation in association with extracellular vesicles (EVs) (see Lettau, M. and Janssen, O. [15] for a recent overview). Notably, EVs were initially described as “platelet dust”, indicating a function as an extracellular disposal system [16] before it was more and more appreciated that they serve as important mediators of intercellular communication [17,18]. EVs are non-replicating vesicles surrounded by a lipid membrane bilayer that are released from almost all cell types and carry a plethora of bioactive proteins, lipids and nucleic acids [19]. They are often categorized based on their size or subcellular origin. Small exosomes (30–150 nm in diameter) originate from multivesicular bodies opening and thus share key processes of biogenesis and respective regulators with LREVs, whereas microparticles/microvesicles (100–1000 nm) are released by plasma membrane blebbing or extrusion. Both types of EVs are constantly released from living cells and are not only important for intercellular communication, but possibly also for exerting effector functions at remote sites. A third group of larger EVs (500–5000 nm) are released as apoptotic bodies from dying cells [15,19].

The aim of the present investigation was to characterize and compare EVs from cytotoxic T cells regarding their protein cargo or surface molecules. Although CD8^+^ TCRαβ^+^ T cells and TCRγδ^+^ T cells are differentially activated in an MHC-dependent (TCRαβ^+^) or MHC-independent (TCRγδ^+^) manner, we know that they store and utilize very similar or even identical combinations of effector proteins for target cell lysis. In this context, we have shown that, for example, in LREVs from expanded healthy CD4^+^ or CD8^+^ TCRαβ^+^ T cells and from TCRγδ^+^ T cells, the unprocessed 15 kDa granulysin is commonly detected in low-density vesicles associated with FasL, while the mature 9 kDa form is more abundant in heavier vesicles [14]. Strikingly, the reported Western blot or imaging analyses suggested a very high degree of similarity regarding effector protein distribution, especially between polyclonally expanded CD8^+^ TCRαβ^+^ T cells and TCRγδ^+^ T cells [14,20].

To get an impression of the composition and the similarity of the protein content of EVs from CD8^+^ TCRαβ^+^ T cells and TCRγδ^+^ T cells, we initially tested the impact of short-term stimulation on EV release. We noted that although stimulation with TPA and ionomycin significantly augmented the release of vesicles, this increase was accompanied by significant activation-induced cell death, especially in zoledronate-expanded γδ T cells. To address the homogeneity of EV preparations from unstimulated and stimulated cells, the enriched vesicles were routinely analyzed by nanoparticle tracking analyses (NTAs). We noted that although stimulation induced EV production, there was no significant change in vesicle size.

For our proteomics analysis, we decided to collect EVs which were constitutively released from growing CD8^+^ αβ T cells and γδ T cells over a final culture period of 72 h. Enriched EVs were lysed and proteins were isobarically labeled with TMT10plex tandem mass tags and analyzed by mass spectrometry. We found that the two distinct cytotoxic T-cell populations share a large set of similarly abundant proteins, while fewer proteins present higher abundance levels in either population.

## 2. Materials and Methods

### 2.1. Cells

All cells were kept at 37 °C in a humidified atmosphere with 5% CO_2_ in RPMI 1640 culture medium with 2 mM glutamine and 25 mM HEPES, supplemented with 100 U/mL penicillin, 100 μg/mL streptomycin and 5–10% (*v*/*v*) fetal bovine serum (FBS) (Thermo Fisher Scientific, Waltham, MA, USA). Peripheral blood mononuclear cells (PBMC) were isolated from blood concentrates of healthy donors provided by the Institute for Transfusion Medicine of the UKSH Campus Kiel by density centrifugation on Ficoll gradients (Merck, Darmstadt, Germany). Written consent of blood donors and an approval by the Institutional Ethics Review Board of the Medical Faculty of Kiel University (file number D467/18) were received to conduct the studies. CD8^+^ T cells were MACS-purified from PBMC using a CD8^+^ T cell isolation kit (Miltenyi Biotech, Bergisch-Gladbach, Germany) following the manufacturer’s guidelines. Positively enriched CD8^+^ cells were 95.8–99.6% CD8^+^ (mean 98.37%, n = 7) and contained 85–98% CD3^+^ T cells, mostly with αβ TCRs and only 2–3% with γδ TCRs. Notably, 5.4–12.4% of the CD8^+^ cells were CD3^−^ and CD56^+^ putative NK cells. After expansion on a feeder cocktail of irradiated EBV-transformed B lymphoblastoid cells and freshly prepared allogeneic PBMC in the presence of 0.5 μg/mL phytohemagglutinin A (PHA, Thermo Fisher Scientific) and rIL-2 (100 U/mL, Novartis, Basel, Switzerland), this phenotype remained stable with a mild increment of CD8^+^ T cells compared to CD56^+^ cells over time. The γδ T cells (i.e., Vδ2 T cells) were enriched by stimulation of 10^8^ PBMC with 1 µg/mL Zoledronate (Novartis) and propagation in RPMI 1640 culture medium supplemented with 50 U/mL rIL-2. During expansion of both cell types, dead cells were removed whenever necessary and fresh medium and rIL-2 were added at least every 3–4 days. After 11–16 days of culture, the zoledronate-stimulated cells were expanded from around 5% in PBMC to more than 93–95% γδ TCR^+^ cells (Supplementary Figure S1).

### 2.2. Immunofluorescent Staining and Flow Cytometry

Phenotyping of the T cells was performed directly after MACS isolation (CD8^+^) or when indicated before the cells were processed for EV preparations. 10^5^ cells were transferred to V-bottom plates and washed once before they were stained with PE-labelled anti-CD3 mab (clone SK7; BioLegend, San Diego, CA, USA), and FITC-labeled anti-CD4 (clone SK3), anti-CD8 (clone SK1), anti-CD56 (clone NCAM16.2), anti-αβ TCR (clone IP26) or anti-γδ TCR (clone 11F2) antibodies (all from BD Biosciences, Franklin Lakes, NJ, USA) for 30 min. After two additional washes and fixation in 1% PFA, samples were analyzed on a FACS Canto flow cytometer (BD Biosciences).

### 2.3. Gel Electrophoresis and Western Blot

Cells were lysed on ice in NP-40 lysis buffer with protease and phosphatase inhibitors and after 20 min of incubation, centrifuged at 4 °C and 14,000 rpm for 10 min to remove cell debris. For gel electrophoresis on Bis-Tris NuPAGE^®^ gels (Thermo Fisher Scientific), 2 µg of protein was boiled in sample buffer without or with β-mercaptoethanol. Since the protein concentration of EV samples was hard to quantify using the standard Bradford assay, 2–5 µL of EV preparations were suspended in NP-40 lysis buffer and processed for gel electrophoresis. Separated proteins were then transferred to nitrocellulose membranes (GE Healthcare, Munich, Germany) and membranes were blocked with 5% (*w*/*v*) bovine serum albumin (Sigma-Aldrich) in TBST. For staining, we used anti-CD81 (5A6) and anti-granulysin (DH2) antibodies from BioLegend with respective HRP-conjugated secondary antibodies from GE Healthcare for detection with ECL chemiluminescence reagents and visualization on Hyperfilm ECL (GE Healthcare).

### 2.4. Preparation of Extracellular Vesicles

To enrich cell-derived EVs, we minimized the EV content in respective culture media by ultracentrifugation. RPMI 1640 medium supplemented with 20% fetal calf serum (FCS) Gibco^®^ (Thermo Fisher Scientific) and penicillin/streptomycin was centrifuged at 100,000× *g* for 18 h. The EV-depleted supernatant was diluted with RPMI 1640 medium to a final concentration of 10% FCS. To obtain extracellular vesicles (EVs) from CD8^+^ αβ T cells or γδ T cells, the cells were depleted from dead cells by Ficoll density centrifugation at day 12–14 of expansion. To investigate the impact of short-term stimulation on the EV release, 4 × 10^8^ vital cells were stimulated or not with the phorbol ester 12-O-tetradecanoylphorbol-13-acetate (TPA at 20 ng/mL) (Merck, Darmstadt, Germany) and calcium ionophore (ionomycin at 500 ng/mL, Merck) at a density of 4 × 10^6^ cells/mL for two additional hours in EV-minimized RPMI 1640 culture medium with IL-2. At the end of the incubation period, cell vitality was determined by dye exclusion using far red dead cell stain (Thermo Fisher Scientific). To prepare constitutively released EVs (e.g., for proteomics analyses), 4 × 10^8^ vital cells were incubated for additional 72 h in 100 mL EV-reduced culture medium with IL-2. EVs were enriched by differential centrifugation and ultracentrifugation as described before [21,22]. Briefly, culture supernatants were centrifuged for 10 min at 300× *g*, 30 min at 2000× *g* and for 45 min at 10,000× *g* to remove intact cells and cell debris. EVs were pelleted by ultracentrifugation at 100,000× *g* for 1.5 h, washed once with filtered (0.1 nm) PBS at 100,000× *g* for 1.5 h and resuspended in 200 µL filtered PBS. EV size and concentration were determined by nanoparticle tracking analysis (NTA) with a NanoSight 300 (NS300) using NanoSight software version 3.40 (Malvern Panalytical, Malvern, UK). For proteome analysis, four individual samples of constitutively released EVs were collected from expanded CD8^+^ αβ T cells and four samples from zoledronate-expanded γδ T cells. The number of particles in individual samples is given in Table 1.

### 2.5. LC-MS-Based Proteome Analysis

EV samples were provided in 200 µL PBS buffer, SDS was added to a final concentration of 1% *w*/*v* and the mixture was supplemented with 1× cOmplete^™^ protease inhibitor cocktail (Roche, Basel, Switzerland). Samples were lysed in a Bioruptor Pico (Diagenode, San Diego, CA, USA) at 4 °C (10 × 30 s on, 30 s off) and 20 µL was used for BCA analysis according to the manufacturer’s protocol (Pierce BCA Protein Assay Kit, Thermo Fisher Scientific). Samples were subjected to reduction and alkylation (12 mM Tris(2-carboxyethyl)phosphine, 40 mM 2-chloroacetamide for 1 h at 25 °C) and 10 µg protein removed for desalting according to the SP3 protocol [23]. SP3 beads were added to each sample (1:40 *w*/*w* protein to beads ratio), then ethanol to 50% *v*/*v* to induce protein binding (10 min at 25 °C). Beads were immobilized using a magnetic rack, supernatants were discarded and the beads washed three times with 80% *v*/*v* ethanol. Pellets were resuspended in 50 mM TEAB buffer (pH 8.5) containing 0.2 µg trypsin (1:50 enzyme to protein ratio, Promega, Madison, WI, USA) and incubated for 16 h at 37 °C. Supernatants were subjected to isobaric labeling with TMT10plex reagent according to the manufacturer’s protocol using eight reaction channels (Thermo Fisher Scientific); labeling scheme: EVs of CD8^+^ αβ T cells: TMT-126, 127N, 127C, 128N; EVs of γδ T cells: TMT-128C, 129N, 129C, 130N (reagents TMT-130C and 131 were intentionally left unused). Samples were combined, solid-phase extraction performed on Pierce C18 pipette tips (Thermo Fisher Scientific) according to the manufacturer’s protocol and samples dried in a vacuum concentrator (Eppendorf, Hamburg, Germany). Dried peptides were resuspended in loading buffer (3% ACN/0.1% TFA in water) and subjected to mass spectrometry analysis.

Samples were injected in quadruplicates on a Dionex Ultimate 3000 nano-UHPLC coupled to a Q-Exactive HF mass spectrometer (Thermo Fisher Scientific). Per injection, 1 µg protein was loaded onto a trap column (Acclaim Pepmap 100 C-18, 5 mm × 300 μm, 5 μm, 100 Å, Dionex, Sunnyvale, CA, USA) and washed for 4 min with 3% ACN/0.1% TFA at a flow rate of 30 μL/min prior to peptide separation using an Acclaim PepMap 100 C-18 analytical column (50 cm × 75 μm, 2 μm, 100 Å, Dionex). A flow rate of 300 nL/min using eluent A (0.05% formic acid (FA)) and eluent B (80% ACN/0.04% FA) was used for gradient separation (5–40% eluent B, 120 min). A spray voltage of 1.8 kV was applied via a liquid junction with a source temperature of 250 °C. Full scan MS spectra were acquired between 300 to 2000 *m*/*z* at a resolution of 60,000 at *m*/*z* 200, and the top ten most intense precursor ions selected for MS/MS analysis (charge state: ≥+2, isolation window: ±1.2 *m*/*z*, HCD fragmentation: 34 NCE). MS/MS spectra were acquired at a resolution of 45,000 with fixed first mass at *m*/*z* 100 and dynamic exclusion list of 40 s.

MS raw files were processed by Proteome Discoverer (version 2.2.0.388, Thermo Fisher Scientific) using the Sequest HT algorithm against a human protein database with additional common contaminating proteins (only reviewed sequences) from the UniProt database [24]. Enzymatic processing was set to trypsin (full-specific), maximum missed cleavage events: 2, minimal peptide length: 6 AA, precursor mass tolerance: 10 ppm, fragment mass tolerance: 0.02 Da and false discovery rate set to q = 0.01 by Percolator node [25]. Modifications were set to oxidation on methionine (+15.995 Da, variable), carbamidomethyl on cysteine (+57.021 Da, static) and TMT reagent on lysine and peptide N-termini (+229.163 Da, static). Quantification was performed by a reporter ions quantifier node (integration tolerance: 10 ppm, average reporter S/N threshold: 10). Results of the first three technical injections were utilized to create a precursor exclusion list for the fourth injection by proteome discoverer (top 5000 entries, retention time tolerance: ±4 min). To verify labeling reactions, TMT over- and under-labeling was quantified.

Results of all four replicates were exported and ratio compression corrected according to Savitski et al. [26]. PSMs were filtered (isolation interference ≤ 50%, all quantification channels present), summarized to protein level (high confidence identifications, minimum two peptides/one unique peptide identification) and data median normalized. Statistical testing was performed by two-sided Welch *t*-test with permutation-based FDR calculation (q = 0.01) in Perseus [27]. Additionally, GO and KEGG annotations were added for 1D enrichment analysis, as well as a Fisher’s exact test against the human proteome FASTA file (both Benjamini–Hochberg FDR, q = 0.01). All proteomics raw data have been uploaded to the ProteomeXchange Consortium [28] via the PRIDE partner repository with the dataset identifier PXD055377.

## 3. Results

### 3.1. Activation Induces the Release of Small Extracellular Vesicles (EVs) from CD8^+^ αβ T Cells and γδ T Cells

All mammalian cells release extracellular vesicles (EVs), either constitutively or upon activation. We noted before that cytotoxic effector molecules or enzymes such as DPP4 (CD26), which are stored and transported in lysosome-related effector vesicles (LREVs), might be released from cytotoxic lymphocytes into the culture supernatant as soluble molecules or in association with EVs [14,15,20]. Constitutively released EV populations should primarily consist of small to medium sized exosomes or microvesicles, and, if the cells remain vital, should not be contaminated by larger proportions of apoptotic bodies. Depending on the strength of stimulation, however, the induction of activation-induced cell death might be associated with a rapid release of apoptotic bodies which presumably also differ in their biogenesis and molecular composition. We therefore enriched constitutively released EVs or EVs produced after short-term activation from cell culture supernatants of CD8^+^ αβ or γδ T cells and characterized them with respect to particle size and concentration via nanoparticle tracking analyses (NTAs).

**Figure 1:**
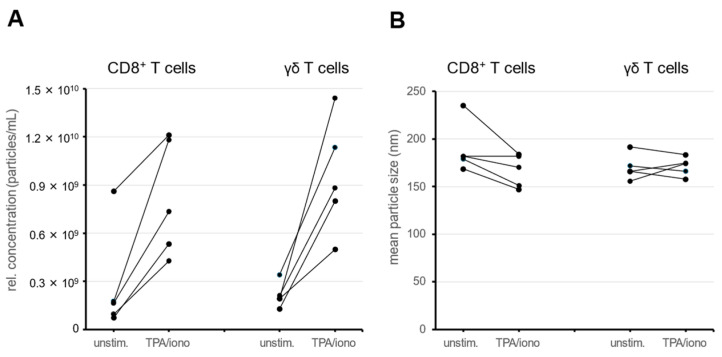
CD8^+^ αβ T cells or γδ T cells were left unstimulated (unstim.) or stimulated for 2 h with phorbol ester and ionomycin (TPA/iono), before EVs were isolated by differential centrifugation and ultracentrifugation and the particle concentration (**A**) and particle size (**B**) were analyzed by NTA. Data for five individual experiments with CD8^+^ αβ T cells or γδ T cells are shown. If less than 4 × 10^8^ cells were plated, the particle concentration was extrapolated respectively for unstimulated and stimulated cells.

The NTA measurements revealed that both CD8^+^ αβ T cells and γδ T cells constitutively release EV particles into the culture supernatants. Stimulation via phorbol ester and calcium ionophore facilitated the release of EVs from both cytotoxic subpopulations and significantly increased the EV concentration in culture media after 2 h of stimulation (Figure 1A). Although we obtained variable results, we observed increased EV production for both populations in all individual experiments and we did not observe substantial differences between αβ T cells and γδ T cells. The relative particle concentration in supernatants of TPA/ionomycin-stimulated αβ T cells was up to seven times higher. Similarly, in γδ T cells, the increment was up to 6.8 fold.

The NTA allowed us to check in parallel whether stimulation would influence the particle size. The mean particle size determined by NTA varied in individual experiments between 145 and 190 nm and did not change significantly with stimulation (Figure 1B). The high value (mean size 235.5 nm) in a single experiment for unstimulated EVs from unstimulated CD8^+^ αβ T cells was rather due to an unusual scattering of two of the four individual NTA measurements used for NTA data calculation.

**Figure 2:**
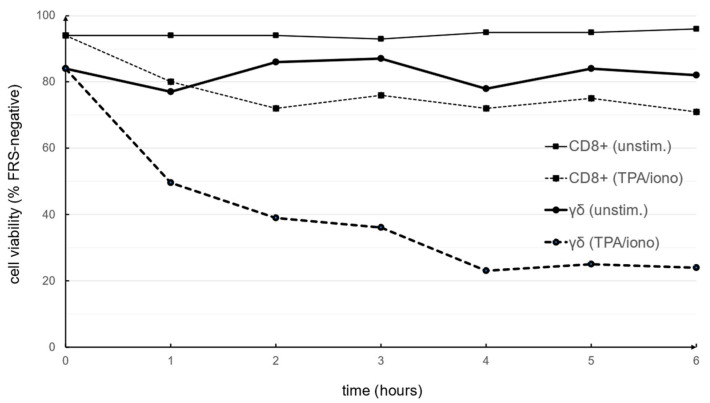
PHA-expanded CD8^+^ αβ T cells (squares and thin lines) and zoledronate-expanded γδ T cells (circles and thick lines) were left untreated or exposed to TPA and ionomycin for six hours. Every hour, cell viability was determined by flow cytometry using far red stain (FRS) exclusion.

**Figure 3:**
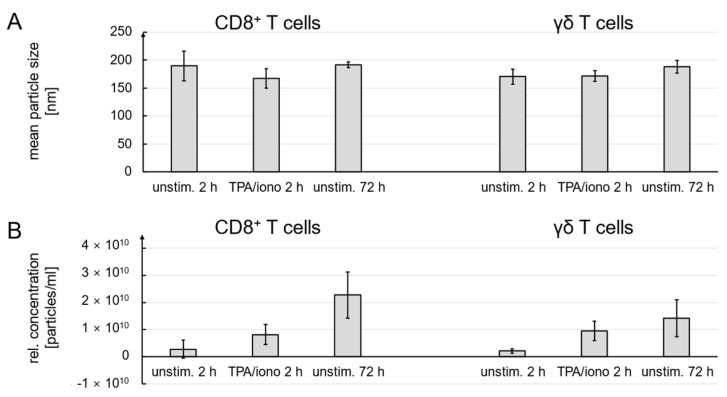
(**A**) Mean particle size. (**B**) Relative particle concentration. CD8^+^ and γδ T cell-derived EVs were subjected to NTA with four individual track measurements for each sample. The cells were stimulated or not with TPA and ionomycin for 2 h (n = 5 for CD8^+^ and n = 5 for γδ^+^ T cells). Alternatively, cells were left untreated for 72 h (n = 8 for CD8^+^ and n = 7 for γδ^+^ T cells). If less than 4 × 10^8^ cells were plated, the particle concentration was extrapolated accordingly.

It is important to consider that strong stimuli might also trigger significant cell death. Interestingly, in a respective control experiment, the induction of cell death by AICD was less prominent in PHA-expanded CD8^+^ αβ T cells. While 96% of the cells were viable after six hours in medium, the viability of CD8^+^ αβ T cells exposed to TPA/ionomycin was reduced to 72% after two hours and remained quite stable over the incubation period. In zoledronate-expanded γδ T cells, however, TPA/ionomycin exposure reduced viability from 82% to 49.5% after two hours and to only 24% after six hours (Figure 2). Given this quite significant reduction in cell viability upon activation and the possible release of larger apoptotic bodies from dying cells, we also compared the particle size and concentration of constitutively released EVs after 72 h of culture in EV-reduced medium in the presence of IL-2. The mean particle size of constitutively released EVs from CD8^+^ αβ T cells was 191.5 nm (n = 8) and from γδ T cells 187.8 nm (n = 7) and was in the same range as particles collected after 2 h with or without stimulation. Standard deviations were less than 10% and all NTA measurements were done with four technical measurements each (Figure 3A, Supplementary Figure S2).

Notably, 72 h of culture in EV-reduced medium in the presence of IL-2 yielded the highest numbers of EVs in individual preparations without inducing activation-induced cell death (Figure 3B). As exemplified in Supplementary Figure S2, all individual EV preparations looked quite homogeneous and monodisperse. EVs from unstimulated cells, harvested two hours after transfer to EV-reduced medium with IL-2, occasionally revealed some additional small peaks for vesicles up to 500 nm which were less prominent in EVs from cells that were stimulated for 2 h or grown for 72 h without stimulation (Supplementary Figure S2). Notably, also for other cell types, the enrichment procedure by differential centrifugation and ultracentrifugation seems to favor the selection of small vesicles and rather exclude larger particles such as apoptotic bodies [21,22].

For the analysis of EV markers and intravesicular proteins, we performed Western blot analyses. Enriched EVs were lysed in NP40 buffer, heated in suited sample buffers and loaded on Bis-Tris NuPAGE^®^ gels for protein separation prior to transfer to nitrocellulose membranes. Following the recommendation by the international society for extracellular vesicles (ISEV), at least two EV markers should be verified as positive controls. As EV markers, we thus tested for CD63 and CD81 and we checked whether the cytotoxic effector protein granulysin was detectable in the enriched EVs. In samples of both CD8^+^ and γδ T cells, the tetraspanin CD63 could be detected in EV samples and in the cell lysates under non-reducing conditions as the typical broad smear with a slight accumulation in EVs. As shown in Supplementary Figure S3, CD81 was detectable in cell lysates, but showed a significant enrichment in EV samples from both cell types. Interestingly, also for the 15 kDa variant of granulysin [14], we noted an accumulation in EVs of γδ T cells and much more prominent in EVs of CD8^+^ T cells.

### 3.2. Comparative Proteome Profiling of Small Extracellular Vesicles (EVs) from CD8^+^ αβ T Cells and γδ T Cells

To gain insight into the molecular composition of constitutively released EVs and to check for potential T-cell subpopulation-specific differences, CD8^+^ and γδ T cell-derived EVs were subjected to liquid chromatography mass spectrometry (LC-MS)-based analysis for proteomic profiling. Notably, the EVs were enriched from MACS-separated and polyclonally activated CD8^+^ cells or zoledronate-stimulated γδ T cells. The cells were expanded for 12 days in the presence of IL-2 and transferred to exosome-depleted medium for 3 more days, before the EVs were enriched by differential centrifugation and ultracentrifugation. The particle concentrations and particle sizes of four individual preparations for each cell type were comparable. Notably, the particle concentration did not correlate to the amount of protein determined in BCA assays (Table 1).

All samples were labelled individually with TMT10plex reagents, and a quantitative bottom-up proteomics analysis was performed on a Q-Exactive HF mass spectrometer. 919 proteins were identified with high confidence in all samples (see Supplementary Table S1), of which 686 proteins were quantifiable (at least 2 peptide/1 unique peptide identifications). Over-labeling (<3.3% of all PSMs) and under-labeling (<0.9% of all PSMs) were neglectable.

**Figure 4:**
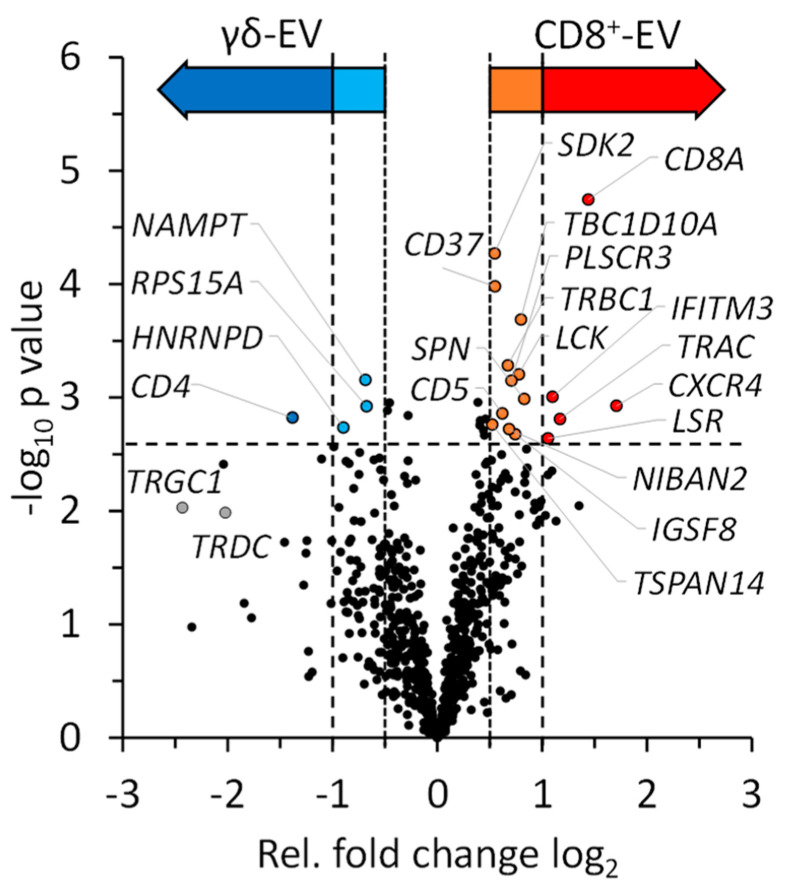
Quantitative analysis of EVs from expanded CD8^+^ T cells and zoledronate-stimulated γδ T cells. Individual proteins with significant fold-change differences are marked and indicated by their gene names within the volcano plot. In addition, we highlighted the TCR γ and δ chains although their fold-change difference did not reach significance. The color code of the arrows corresponds to Supplementary Table S2: Dark Red – Significantly higher abundant in EVs of CD8^+^ T cells (Strict); Light Red—Significantly higher abundant in EVs of CD8^+^ T cells (Moderate); Dark Blue—Significantly higher abundant in EVs of γδ T cells (Strict); Light Blue—Significantly higher abundant in EVs of γδ T cells (Moderate).

The quantitative analysis revealed a significantly higher abundance in EVs of CD8^+^ T cells for the C-X-C chemokine receptor type 4 (*CXCR4*), the T-cell surface glycoprotein CD8 alpha chain (*CD8A*), the T cell receptor alpha constant (*TRAC* = *TCRA*), the interferon-induced transmembrane protein 3 (*IFITM3*) and the lipolysis-stimulated lipoprotein receptor (*LSR = LISCH*). Other proteins that were significantly more abundant in the EVs of CD8^+^ T cells included leukosialin (CD43, *SPN*), the tyrosine-protein kinase p56lck (*LCK*), the T cell receptor beta constant 1 (*TRBC1*), the T-cell surface glycoprotein CD5, the sidekick cell adhesion molecule 2 (*SDK2*) and the tetraspanins 26 (*CD37*) and 14 (*TSPAN14*). Moreover, PI-kinase alpha and the proform of IL-16 were significantly more abundant in the EVs of CD8^+^ T cells and Flotillin, RhoF and Moesin were also higher, but not significantly (Figure 4 and Supplementary Table S2).

CD4 (*CD4*) was identified as being significantly more abundant in zoledronate-expanded γδ T cells than in MACS-sorted and expanded CD8^+^ T cells. This is most probably due to the co-expansion of a few (maximum 5–10%) CD4^+^ T cells during the zoledronate-driven expansion, which were completely lacking in MACS-purified CD8^+^ T cells. The 40S ribosomal protein S15a (*RPS15A*), the nicotinamide phosphoribosyltransferase (*NAMPT*) and the heterogeneous nuclear ribonucleoprotein D0 (*HNRNPD*) were significantly more abundant in EVs of zoledronate-stimulated γδ T-cells (Figure 4). As expected, the T-cell receptor gamma chain C region 1 (*TRGC1*) and the T-cell receptor delta chain C region (*TRDC*) were more abundant in zoledronate-expanded cells, although not significant (Figure 4). The list of proteins with not significant but biologically relevant FC differences also included the C-X-C chemokine receptor type 6, granzyme B and granulysin, annexin A4 and several histones (Supplementary Table S2).

**Figure 5:**
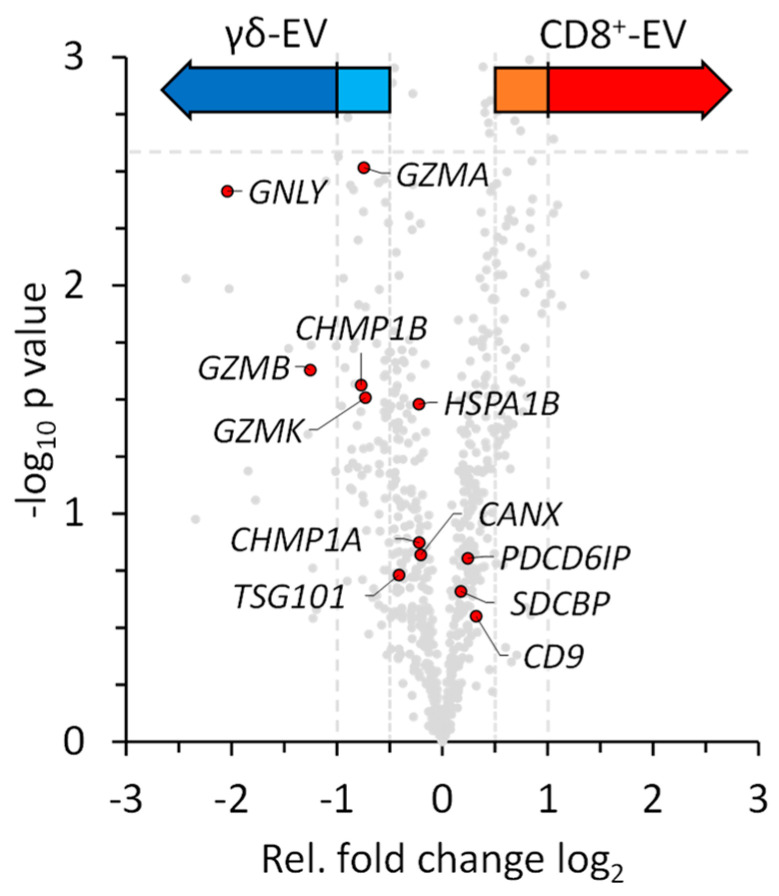
Quantitative analysis of EVs from expanded CD8^+^ T cells and zoledronate-stimulated γδ T cells. Individual EV-associated markers and effector proteins are highlighted and indicated by their gene names within the volcano plot. Color code as described in Figure 4.

As expected, the mass spectrometry data revealed the presence of EV-specific markers and effector proteins (Figure 5). The CD9 antigen (*CD9*) was identified and quantified, CD81 was identified, but not quantified and CD63 was not identified due to lacking significance according to the FDR calculation. The 70 kDa heat shock protein 1B (*HSPA1B* = *HSP70-2*), which is often identified in EVs from different cell types, and proteins associated with multivesicular body biosynthesis including syntenin-1 (*SDCBP*), the tumor susceptibility gene 101 protein (*TSG101*) and the programmed cell death protein 6 (*PDCD6IP* = *ALIX*) were quantifiable but did not show differences in abundance in the EV preparations (Supplementary Table S1, Figure 5).

In addition, as components of the ESCRT machinery, the charged multivesicular body protein 1a (*CHMP1A*) and 1b (*CHMP1B*) were identified. Surprisingly, calnexin (*CANX*) was also quantifiable, although it is regarded as a negative marker for exosomes. Intravesicular effector proteins such as granulysin (*GNLY*) and granzymes A, B and K (*GZMA*, *GZMB*, *GZMK*) were quantifiable and collectively more prominent in zoledronate-expanded T-cells (Figure 5). As mentioned before, proteins that could not be quantified in the mass spectrometry analysis include CD63 and CD81.

Interestingly, of the four biological replicates, only one showed a slightly different ratio calculation plot, indicating high homogeneity of the prepared samples (see Supplementary Figure S4).

**Figure 6:**
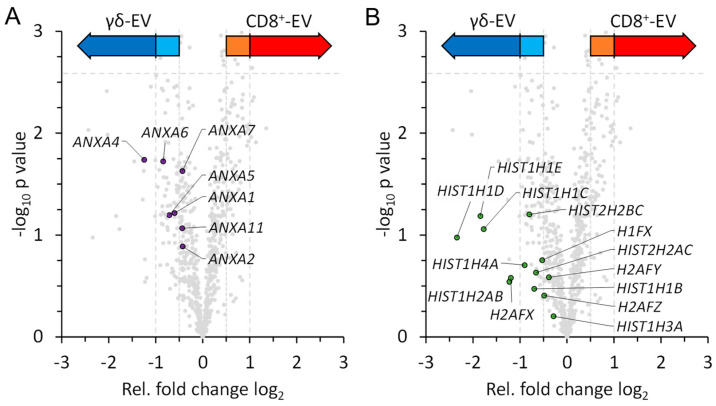
Quantitative analysis of EVs from expanded CD8^+^ T cells and zoledronate-stimulated γδ T cells. Individual annexins (**A**) and histones (**B**), which were more abundant in EVs from γδ T cells, are highlighted and indicated by their gene names within the volcano plot. Color code as described in Figure 4.

All-in-all, we observed a widely homogeneous protein content of EVs from the two T-cell populations. Nonetheless, we noted interesting differences in the relative abundance of various annexins (i.e., annexin A1 (*ANXA1*), A2 (*ANXA2*), A4 (*ANXA4*), A5 (*ANXA5*), A6 (*ANXA6*), A7 (*ANXA7*) and A11 (*ANXA11*)) which, although not significant, were more prominent in EVs from zoledronate-expanded T-cells (Figure 6A). Moreover, these vesicles also contained higher abundance levels of several histones (e.g., histones H1.2 (*HIST1H1C*), H1.3 (*HIST1H1D*), H1.4 (*HIST1H1E*), H2A type 1 (*HIST1H1C*), H2AX (*H2AFX*) and many others) which are visible in the lower left area of the volcano plot (Figure 6B). This effect is also reflected within the 1D enrichment analysis, which highlights significantly higher abundances of proteins associated with nucleic acid binding within the EVs of γδ T cells (Supplementary Table S3).

Additionally, we analyzed our list of protein identifications against the human proteome to identify enriched GO terms within the entire dataset (Supplementary Table S4). This Fisher’s exact test provided numerous terms, with the top five terms by FDR level being extracellular vesicular exosome (GO:0070062), extracellular membrane-bound organelle (GO:0065010), extracellular organelle (GO:0043230), membrane-bound vesicle (GO:0031988) and vesicle (GO:0031982). Enrichment factors of these terms reached from 4.565 to 3.934, which strongly supports the presence of small EVs/exosomes.

## 4. Discussion

### 4.1. Production and Initial Characterization of Small Extracellular Vesicles (EVs) from CD8^+^ αβ T Cells and γδ T Cells

To characterize the EVs of cytotoxic CD8^+^ αβ T cells and γδ T cells, we used an approved centrifugation and ultracentrifugation protocol adapted in our laboratory primarily for the enrichment of exosomes and microvesicles from cell culture supernatants of malignant cells [21,22]. To reduce the amount of EV intake from media supplements such as FBS, the culture media were centrifuged with 100,000× *g* over 18 h and supernatants filtered with a 0.1 nm filter prior to use. Importantly, in our study we compared the activation-induced EV production and the short- or long-term release of EVs from both cell types. However, in the end we decided to analyze EVs which were constitutively released from not further stimulated cells over a final culture period of 72 h in an EV-depleted medium in the presence of IL-2. This is because we are fully aware of the fact that all biological manipulations that might be intended to increase EV production might also affect EV biogenesis and result in biologically altered vesicles [29]. Additionally, the mechanisms leading to increased EV production in individual cell populations are still widely unknown. In fact, it is believed that EVs are inherently heterogeneous, with great donor to donor variations regarding size and cargo, and that further manipulation of the cells of origin might even enhance heterogeneity [29]. We rather follow the concept that T cell-derived EVs share important steps of biogenesis with intracellular LREVs [15], and we do not agree with the idea of distinct heterogeneity. In this context, we have shown earlier that LREVs from expanded untransformed T cells of different donors are indeed very homogeneous [8]. They of course carry individual MHC molecules, but their overall protein content is close to identical [30].

Activation of expanded T cells in vitro is usually associated with transcriptional activity (e.g., resulting in increased expression of FasL [31,32]) that definitely leads to altered protein loading of intracellular LREVs and consequently also to alterations in the content and composition of EVs. Moreover, activation might induce degranulation and we know that differential signaling is associated with differential degranulation [14,20]. In our present study, we demonstrate that the rather strong stimulation with calcium ionophores and phorbol ester resulted in a significant increase in EV release from both cell populations compared to unstimulated controls (Figure 1).

We also determined by NTA that the size of the enriched vesicles did not change significantly whether cells were stimulated or not. Although this is in line with previous observations of other groups, claiming that MVB fusion to cell membranes requires Ca^2+^ signals [33,34], we decided to also consider cell death induction by TPA and ionomycin as an important parameter for vesicle production, especially for the release of apoptotic bodies from dying cells. In fact, in our own earlier studies on AICD, we often used TPA and ionomycin as a positive control for maximal induction of cell death in αβ and γδ T-cell populations [35,36]. Notably, the induction of cell death by apoptosis is associated with an increased production of apoptotic bodies from dying cells, which—as a third form of EVs—might contaminate the EVs released as exosomes or microvesicles from vital cells [37]. We therefore analyzed the vitality of unstimulated and stimulated cells and found that TPA/ionomycin induced massive cell death in zoledronate-expanded γδ T cells (and moderate but significant cell death in CD8^+^ αβ T cells). Notably, cells that were not stimulated and further expanded in medium with Il-2 did not reveal any major change in vitality (Figure 2). Moreover, we analyzed the size and concentration of EVs that were constitutively released over a period of 72 h and compared them to EVs liberated after two hours without or with stimulation. Again, we noted no differences in EV size, but a further increase in EV concentrations (Figure 3). Therefore, we decided to collect constitutively released EVs after a final culture period of 72 h in an EV-reduced medium for the intended proteomic profiling.

Although we expanded the cell lines following a rather strict protocol, polyclonal activation resulted in different numbers of cells that could be subjected to EV production and did not always reach exactly 400 × 10^6^ cells. One also must keep in mind that expanded CD8^+^ cells sometimes still include a minor portion of NK or γδ T cells, and zoledronate-stimulated γδ T cells (including those used for the proteomic profiling) might have contained a few co-expanded CD4^+^ αβ T cells and potentially also other γδ subpopulations with different Vδ elements that might produce distinct EVs with different protein loading and functions within the local microenvironment (Supplementary Figure S1). All-in-all, our strategy was to avoid the promotion of AICD by (re-)stimulation and to collect supernatant from a decent number of vital cells that were kept in the presence of IL-2. In terms of EV function, we isolated constitutively produced vesicles which might differ in biosynthesis and mobilization from exosomes that are released from MVBs by Ca^2+^-triggered degranulation.

The NTA-derived parameters indicate that according to the recommended classification of the international society for extracellular vesicles (ISEV), the isolated EVs can be categorized as small extracellular vesicles with a mean particle size of less than 200 nm [24] (Figure 1 and Figure 2). In Western blots, we detected CD81 and granulysin (Figure S1) and the proteomic profiling revealed the presence of numerous expected EV marker proteins including CD63, CD9, syntenin-1, TSG101 and ALIX [38], and T-cell- and subpopulation-specific proteins including CD3 components, individual TCR chains, CD8 and several T-cell-specific signaling proteins (Supplementary Table S2) [39]. Although there is still some debate as to whether EVs represent a fingerprint of the mother cell [40], our data clearly point into this direction. In our experiments, the characteristic TCR components or CD8 chains segregated to the EVs isolated from the respective cell population. Notably, the identification of CD4, which was selectively detected in zoledronate-stimulated cells, is likely due to a mild co-expansion of supporting helper cells from the PBMC.

Since CD8^+^ CTL and cytotoxic γδ T cells play a major role in the elimination of virus-infected and malignant cells, we expected to detect cytotoxic effector molecules in EV preparations from both cell types. Moreover, over the past decade, we heavily investigated the transport and mobilization of cytotoxic effector proteins including FasL, granzymes, perforin and granulysin in T and NK cells [6,13,14,15,20,31,41], and we reported their storage and distribution to different lysosome-related effector vesicles (LREVs) [9,13,14,20]. In fact, we reported that different subtypes of LREVs might be distinguished by the presence of FasL and 15 kDa granulysin, or granzyme B and 9 kDa granulysin, respectively [14]. As expected, granulysin could also be used as a marker for EVs from cytotoxic T cells (Figure S2). Here, the enriched EVs from both populations primarily contained the 15 kDa form of granulysin, indicating that the isolated EVs reflect the light granules which are released in a Ca^2+^-independent fashion [14].

Notably, we previously also found, primarily, the 15 kDa form of granulysin in EV preparations. In an earlier study, we had compared the overall protein content of LREVs with EVs originating from split CD4^+^, CD8^+^ and γδ T-cell blast populations. When individual samples were analyzed by 2D-DIGE, in all T-cell subsets, EVs were consistently more similar to light LREVs. Additionally, more similarities were found between the two types of LREVs than between EVs and dense LREVs, suggesting that light LREVs and exosomes might share more common pathways during biogenesis. Importantly, constitutively released exosomes and exosomes released upon TCR ligation showed a very high degree of similarity (up to 95%) in the 2D-DIGE experiments, with only a few protein spots being increased in the exosomes after stimulation. Assuming that intact LREVs are supposed to exhibit an additional membrane and luminal space, this further substantiated the idea that such EVs might rather represent liberated light LREVs [15].

### 4.2. Quantitative Proteomic Profiling of Small Extracellular Vesicles (EVs) from CD8^+^ αβ T Cells and γδ T Cells

Quantitative bottom-up proteomics was used to compare the proteome of EVs that were constitutively released from cytotoxic CD8^+^ and γδ T cells. Interestingly, both populations delivered comparable amounts of vesicles resulting in comparable amounts of protein that could be used for labeling. Notably, the concentration and size of the EV preparation were determined directly after isolation. Since samples could not directly be processed for mass spectrometry, they were frozen at −20 °C and carefully thawed for further use. Although we have not tested this in detail for the present study, we and others previously noticed that a single freeze-thaw cycle does not significantly reduce the concentration or influence the size (or activity) of isolated EVs [42]. Nonetheless, storage parameters for EVs, such as duration and temperature, were discussed as a topic of concern in the 2023 ISEV meeting [43,44]. A recent study by Gelibter et al. indicated a decrease in EV concentrations after four weeks and after six months at −80 °C [45]. Notably, due to the parallel labeling of the individual EV samples and the processing in four replicates, the influence of vesicle loss due to a single freeze-thaw cycle might be neglected for the purpose of the present study. This is also highlighted by the fact that only one out of four biological replicates showed a slightly broader ratio calculation plot indicating a decent homogeneity of the prepared samples (Supplementary Figure S3).

All in all, in our quantitative bottom-up proteomics analysis, 919 proteins were identified with high confidence in all samples (Supplementary Table S1). Of those, 686 proteins were quantifiable. The quantitative analysis revealed a significantly higher (strict) abundance in EVs of CD8^+^ T cells only for the five proteins CXCR4, CD8 alpha, TCR alpha, the interferon-induced transmembrane protein 3 and the lipolysis-stimulated lipoprotein receptor. Eleven proteins showed significantly higher (moderate) abundance level in EVs of CD8^+^ T cells. These included the TCR beta constant region, CD5, CD37, CD43 and p56lck (Supplementary Table S2).

As mentioned before and to our surprise, CD4 was identified as a single protein being significantly (strict) more abundant in zoledronate-expanded γδ T cells. We assume that this is due to the detection of CD4 on EVs of co-expanded CD4^+^ T cells which were not depleted during the zoledonate-driven expansion. In contrast, CD4^+^ cells were *a priori* lacking in the CD8^+^ population due to the (positive) MACS purification. Interestingly, with the 40S ribosomal protein S15a, the nicotinamide phosphoribosyl transferase and the heterogeneous nuclear ribonucleoprotein D0, three proteins associated with DNA/RNA processing were significantly more abundant in EVs of zoledronate-stimulated γδ T-cells. The higher abundance (although not significant) of parts of the TCR gamma chain and the TCR delta chain C in zoledronate-expanded cells was expected and reassuring. We would like to once more stress that the mass spectrometry data confirmed the presence of EV-specific markers and effector proteins including the tetraspanins CD9 and CD81, the heat-shock protein HSP70 and proteins involved in EV biosynthesis including syntenin-1, TSG101 and ALIX. Notably, most of these proteins were quantifiable but did not show major differences in abundance in the EVs from the two cell populations. As cytotoxic effector proteins, granulysin and the granzymes A, B and K were quantifiable and slightly more abundant in zoledronate-expanded cells (Figure 5). The short lists of differentially abundant proteins indicate that we determined a widely homogeneous protein content of EVs from the two cytotoxic T-cell populations. Nonetheless, we noted surprising differences in the relative abundance of annexins and histones which were more prominent in EVs from zoledronate-expanded T-cells.

One of the open questions is whether the constitutively released EVs analyzed in our study might be categorized as plasma membrane-derived microvesicles rather than MVB-derived exosomes [46]. According to Haraszti and colleagues, the protein patterns of exosomes are more likely to be different depending on their cells of origin than are the protein patterns of microvesicles. In their study, tumor cell-derived exosomes exhibited proteins functionally associated with the extracellular matrix, heparin-binding, receptors, the immune response and cell adhesion, whereas microvesicles were enriched in endoplasmic reticulum, proteasome and mitochondrial proteins [46].

Although not significant, we identified a plethora of proteins that allow for a glance at the multitude of putative functions of EVs in T-cell biology. In line with their proposed accessory function of EVs in target cell killing [15], we found the cytotoxic effector proteins granulysin, granzyme B (both with a tendency to be more abundant in γδ T cells) and perforin, thus equipping EVs with direct tumoricidal properties. In addition to TCR components, T-cell EVs contained all individual CD3 complex subunits as well as signaling adapters like LAT or PAG, kinases like ZAP-70, Fyn and Lck and adapter proteins like Nck supporting the previous notion that T-cell EVs effector functions might retain antigen-specificity to a certain degree [39]. Moreover, we also identified BTN3A3, a surface molecule contributing to the recognition of phosphoantigens [47] and DNAM-1 (CD226), an activating receptor recognizing CD155 and CD112 that are often upregulated on tumour cells. DNAM-1 was shown to increase the binding and/or the internalization of NK cell-derived EVs to tumor cells to directly facilitate apoptosis [48]. CD2 and CD6 also contribute to T-cell adhesion and might additionally serve costimulatory functions [49,50], especially in the context of γδ T cells [51]. Interestingly, we identified CD47 to be associated with T-cell EVs. CD47 is a “don’t eat me” signal for macrophages that is discussed to prolong the half-life of EVs [52] and in this way to allow for a more efficient targeting of malignant cells [53].

We additionally performed a 1D annotation enrichment to identify annotated GO-terms, to which corresponding proteins might not have presented a significant fold change on their own (Supplementary Table S3). Results of this enrichment analysis are split into two groups. Those GO-terms, to which corresponding proteins presented higher fold changes within EVs of γδ T cells, and those to which proteins presented higher fold changes within EVs of CD8^+^ T cells. Within the first group, covering γδ T cells, many GO-terms associated to DNA or RNA binding were identified. This included the terms nucleic acid binding (GO:0003676), ribonucleoprotein complex (GO:0030529), as well as DNA and RNA binding (GO:0003677, GO:0003723). In part, this reflects the previously described identification of multiple histone proteins, which provided increased abundance levels in EVs of γδ T cells (Figure 6). However, it also encompassed many other proteins, as the term nucleic acid binding covered up to 128 proteins in our dataset. On the other hand, GO-terms with higher fold changes in CD8^+^ EVs presented the localization term cell periphery (GO:0071944), as well as multiple terms related to the cytoskeleton and its organization by actin filaments (GO:0007015, GO:0008092, GO:0030036, GO:0007010 and GO:0005856).

Our previous notion about the potential biogenesis of constitutively released EVs as plasma membrane-derived microvesicles might be supported by the fact that the GO-terms membrane part (GO:0044425) and plasma membrane (GO:0005886) covered 258 and 264 proteins, respectively, in our dataset. As a clear indication for the close relationship between intra- and extracellular vesicles [15], very high numbers of identified proteins were covered by the GO-terms intracellular membrane-bound organelle (GO:0043231, 376 proteins), intracellular organelle part (GO:0044446, 404), organelle part (GO:0044422, 410) and intracellular organelle GO:0043229, 447).

Finally, we would like to stress once more that the Fisher’s exact test where we analyzed our list of protein identifications against the human proteome to identify enriched GO terms within the entire dataset provided numerous terms, with the top five terms by FDR level being extracellular vesicular exosome (GO:0070062), extracellular membrane-bound organelle (GO:0065010), extracellular organelle (GO:0043230), membrane-bound vesicle (GO:0031988) and vesicle (GO:0031982) (Supplementary Table S4). The high enrichment factors of these terms clearly indicate the presence of small extracellular vesicles in our preparations. Notably, these terms also do not discriminate between exosomes and microvesicles.

How do the present results fit to the idea of supramolecular attack particles (SMAPs) which was recently brought up by Balint and colleagues [54] and Chang and coworkers [55]? As mentioned before, it has been known for more than 20 years that “secretory lysosomes”, or later termed “lysosome-related effector vesicles” (LREVs), store and transport granzymes and perforin and—maybe more important as a weapon—carry FasL [2,56,57,58]. In fact, we occasionally also observed multivesicular bodies (MVBs) in our preparations of intracellular vesicles, which might have contained different types of vesicles or even supramolecular protein aggregates according to our nomenclature. Presumably, however, such vesicles might have sedimented differentially in the iodixanol gradients used for vesicle enrichment. In our studies, MVBs were quite rare in preparations for EM. Additionally, it is also commonly accepted that a portion of the intracellular storage vesicles originate from MVBs [15]. Of note, the observation that cytotoxic effector molecules are stored in association with the anionic proteoglycan serglycin, that facilitates both storage and effector function upon (co-)release, already hinted at the presence of supramolecular protein aggregates that might form a functional unit [59].

Notably, it has been shown by Ostergaard and colleagues [10,11], by the group of Thomi Brunner [12] and also by us [13,14] that FasL-containing light vesicles need less strong signals (PKC only) for mobilization as compared to dense LREVs which require additional calcium signals. In essence, different signal strengths result in differential release of effector proteins. Notably, these observations were also regarded as an argument for different vesicular entities and this concept might be extended to supramolecular protein aggregates that act as a functional unit besides vesicles.

Interestingly, Chang and colleagues identified two classes of fusion-competent granules, which they called single core granules (SCGs) and multi core granules (MCGs). Both types of granules fuse with the plasma membrane at the immunological synapse. SCGs open and release e.g., soluble GzmB, whereas MCGs release intact SMAPs. In essence, they propose that single core granules release their cytotoxic content instantly into the synapse while in parallel multi core granules fuse with the membrane to deliver latent SMAPs for delayed killing of refractory targets.

From our proteomic profiling, we cannot assign the EVs to either type of granule although 252 common proteins were identified both in our EV preparations and in SMAPs. However, since our EV preparations apparently consist of intact vesicles that carry numerous transmembrane proteins and at the same time granzymes, perforin, granulysin etc., they might represent MCG-derived SMAPs. Notably, the constitutively released extracellular vesicles investigated in our study also carried the SMAP-marker thrombospondin-1. This would, however, mean that expanded T cells constitutively release potentially active bioweapons, and it poses the questions how these weapons find and interact with their remote targets and why there are so many other proteins associated with such SMAPs.

## 5. Conclusions

We provide a comparative analysis of extracellular vesicles (EVs) from activated and expanded cytotoxic CD8^+^ αβ T cells or γδ T cells. Both cell types constitutively release small EVs. EV production can be enhanced by in vitro stimulation, however, at the expense of augmented cell death. We thus collected constitutively produced EVs over a final culture period of 72 h and enriched them by differential centrifugation and ultracentrifugation. The obtained small EVs shared common properties as analyzed by nanoparticle tracking analyses, Western blotting and mass spectrometry. In a quantitative bottom-up proteomics analysis, EV preparations from both cell types were labeled with tandem mass tags and subjected to mass spectrometry analysis. A total of 919 proteins were identified with high confidence in all replicates. Of those, 686 proteins were quantifiable. The two cytotoxic T-cell populations shared a major set of similarly abundant proteins and only a few proteins presented significantly higher abundance levels in either CD8^+^ αβ T cells or γδ T cells. Despite of these overall similarities, the 1D annotation enrichment revealed several GO-terms that were more associated with EVs from one or the other cell population.

## Figures and Tables

**Table 1:** Particle concentrations and protein content of EV preparations. EVs were purified as described, suspended in 200 µL of filtered PBS and analyzed by nanoparticle tracking analysis (NTA) and BCA protein assay.

	Particles/mL (NTA)	Size (nm) Mean/Mode (NTA)	Absorption (BCA)	Protein (µg/µL)	Total (µg)
CD8^+^ EV					
# 1	1.44 × 10^10^ ± 7.37 × 10^8^	189.8/126.9	0.174	0.073	14.5
# 2	1.44 × 10^10^ ± 8.01 × 10^8^	191.8/129.1	0.158	0.060	11.9
# 3	1.77 × 10^10^ ± 4.95 × 10^8^	196.5/158.4	0.231	0.120	23.9
# 4	2.20 × 10^10^ ± 5.97 × 10^8^	197.6/205.7	0.178	0.076	15.2
γδ EV					
# 1	1.89 × 10^10^ ± 1.13 × 10^9^	185.1/127.7	0.169	0.069	13.7
# 2	1.77 × 10^10^ ± 1.38 × 10^9^	176.7/112.8	0.149	0.052	10.4
# 3	1.05 × 10^10^ ± 8.45 × 10^8^	198.8/140.0	0.172	0.071	14.2
# 4	5.45 × 10^9^ ± 3.42 × 10^8^	191.6/162.7	0.146	0.050	9.9

## Data Availability

All proteomics raw data have been uploaded to the ProteomeXchange Consortium [38] via the PRIDE partner repository with the dataset identifier PXD055377.

## Supplementary Materials

The following supporting information can be downloaded at https://www.mdpi.com/article/10.3390/cells13201745/s1, Supplementary Proteomics Tables, containing Table S1: List of high-confidence protein identifications; Table S2: Results of statistical Welch *t*-test with permutation-based FDR, q = 0.01; Table S3: Results of 1D annotation enrichment analysis with Benjamini–Hochberg FDR, q = 0.01; Table S4: Results of Fisher’s exact test with Benjamini–Hochberg FDR, q = 0.01; Figure S1: Phenotype of PHA-expanded CD8^+^ T cells (A) and zoledronate-expanded γδ T cells (B); Figure S2: Representative nanoparticle tracking analyses (NTAs); Figure S3: Detection of EV marker and effector proteins by Western blotting; Figure S4: Histograms depicting the distribution of relative protein fold changes for log_2_(CD8^+^-EV/γδ-EVs).
