# Supplementary material for: Comparative Analysis of Extracellular Vesicles from Cytotoxic CD8+ αβ T Cells and γδ T Cells
Source: Cells. 2024 Oct 21;13(20):1745. doi: 10.3390/cells13201745 (PMC11506423; doi:10.3390/cells13201745)
Supplement: Supplementary file 1 [file cells-13-01745-s001.zip › Supplementary Fig. S4.pptx]

## Slide 1
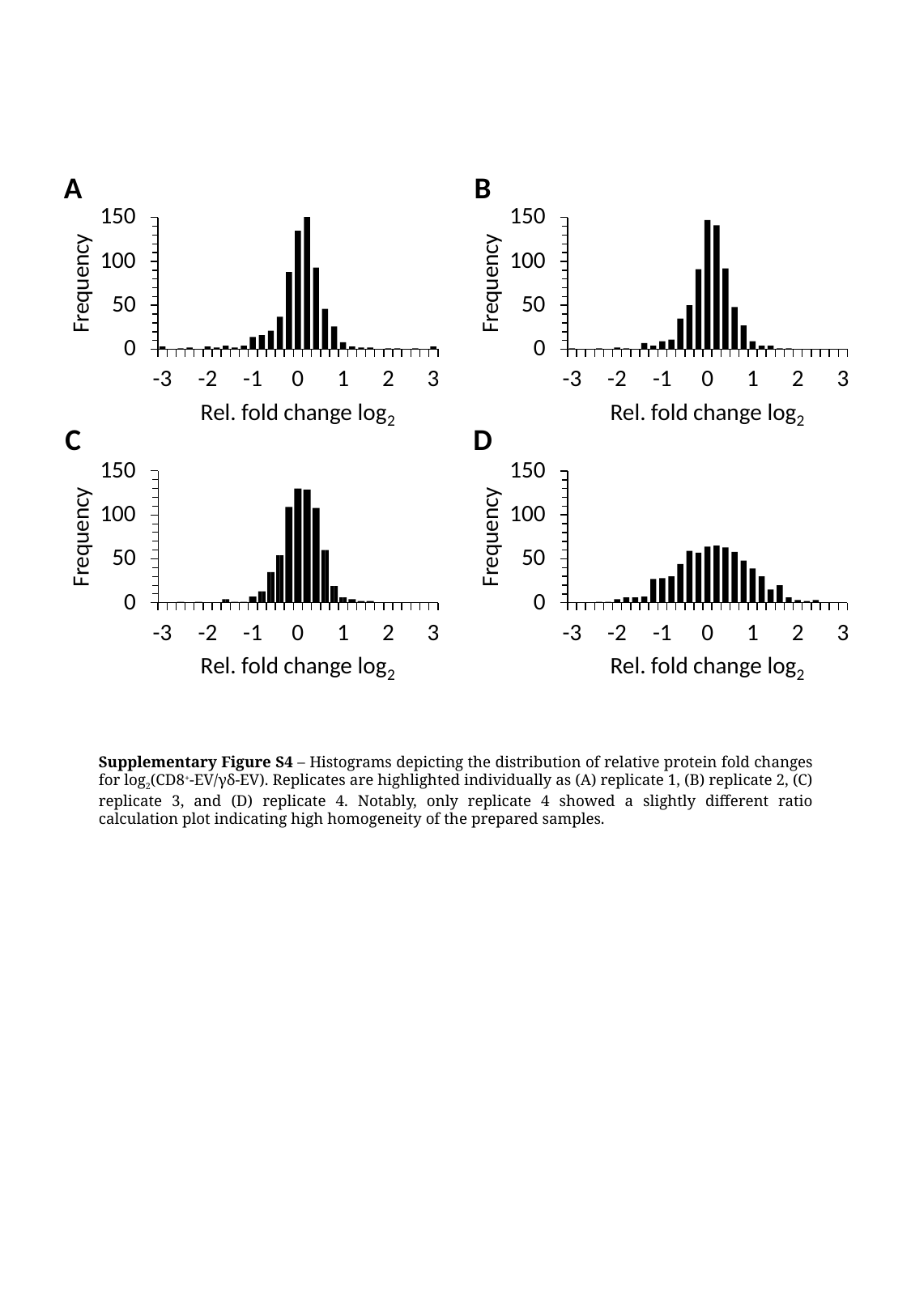

Supplementary Figure S4 – Histograms depicting the distribution of relative protein fold changes for log2(CD8+-EV/γδ-EV). Replicates are highlighted individually as (A) replicate 1, (B) replicate 2, (C) replicate 3, and (D) replicate 4. Notably, only replicate 4 showed a slightly different ratio calculation plot indicating high homogeneity of the prepared samples.
