# Supplementary material for: Comparative Analysis of Extracellular Vesicles from Cytotoxic CD8+ αβ T Cells and γδ T Cells
Source: Cells. 2024 Oct 21;13(20):1745. doi: 10.3390/cells13201745 (PMC11506423; doi:10.3390/cells13201745)
Supplement: Supplementary file 1 [file cells-13-01745-s001.zip › Supplementary Fig. S3.pptx]

## Slide 1
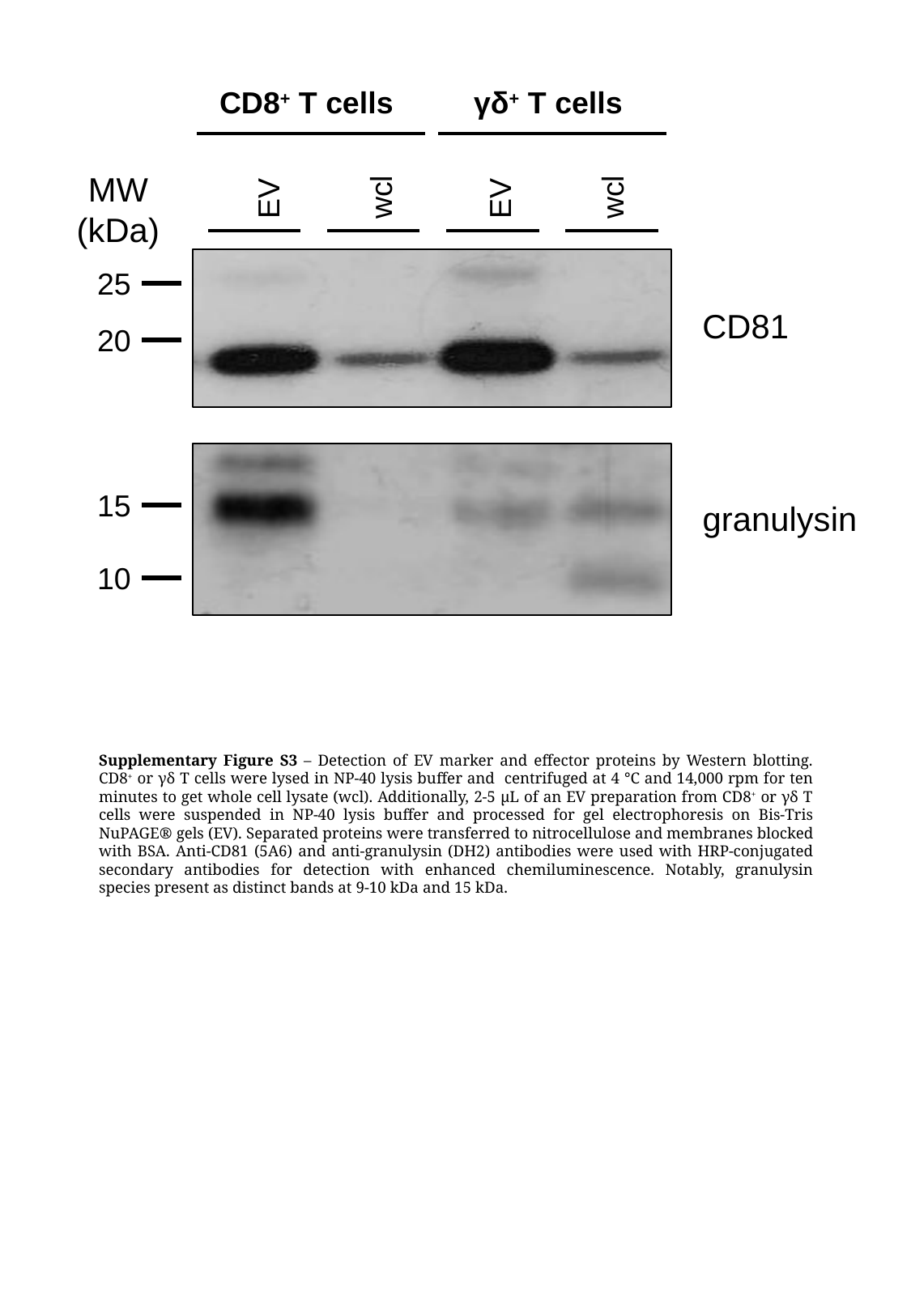

γδ+ T cells
CD8+ T cells
EV
wcl
EV
wcl
MW
(kDa)
25
CD81
20
15
granulysin
10
Supplementary Figure S3 – Detection of EV marker and effector proteins by Western blotting. CD8+ or γδ T cells were lysed in NP-40 lysis buffer and centrifuged at 4 °C and 14,000 rpm for ten minutes to get whole cell lysate (wcl). Additionally, 2-5 µL of an EV preparation from CD8+ or γδ T cells were suspended in NP-40 lysis buffer and processed for gel electrophoresis on Bis-Tris NuPAGE® gels (EV). Separated proteins were transferred to nitrocellulose and membranes blocked with BSA. Anti-CD81 (5A6) and anti-granulysin (DH2) antibodies were used with HRP-conjugated secondary antibodies for detection with enhanced chemiluminescence. Notably, granulysin species present as distinct bands at 9-10 kDa and 15 kDa.
