# Supplementary material for: Comparative Analysis of Extracellular Vesicles from Cytotoxic CD8+ αβ T Cells and γδ T Cells
Source: Cells. 2024 Oct 21;13(20):1745. doi: 10.3390/cells13201745 (PMC11506423; doi:10.3390/cells13201745)
Supplement: Supplementary file 1 [file cells-13-01745-s001.zip › Supplementary Fig. S1.pptx]

## Slide 1
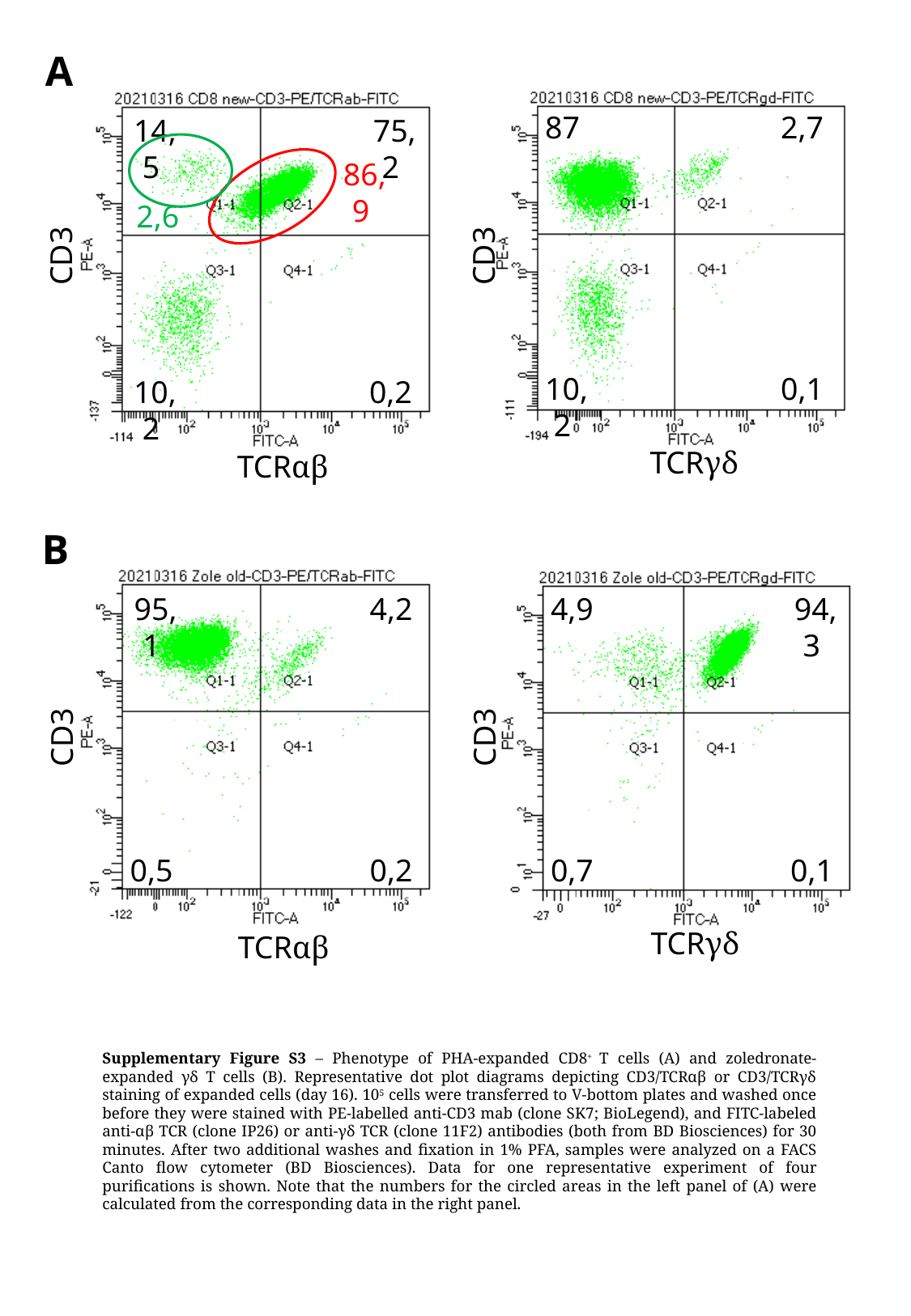

A
87
2,7
14,5
75,2
86,9
2,6
CD3
CD3
10,2
0,1
10,2
0,2
TCRγδ
TCRαβ
B
95,1
4,2
4,9
94,3
CD3
CD3
0,5
0,2
0,7
0,1
TCRγδ
TCRαβ
Supplementary Figure S3 – Phenotype of PHA-expanded CD8+ T cells (A) and zoledronate-expanded γδ T cells (B). Representative dot plot diagrams depicting CD3/TCRαβ or CD3/TCRγδ staining of expanded cells (day 16). 105 cells were transferred to V-bottom plates and washed once before they were stained with PE-labelled anti-CD3 mab (clone SK7; BioLegend), and FITC-labeled anti-αβ TCR (clone IP26) or anti-γδ TCR (clone 11F2) antibodies (both from BD Biosciences) for 30 minutes. After two additional washes and fixation in 1% PFA, samples were analyzed on a FACS Canto flow cytometer (BD Biosciences). Data for one representative experiment of four purifications is shown. Note that the numbers for the circled areas in the left panel of (A) were calculated from the corresponding data in the right panel.
