# Supplementary material for: Comparative Analysis of Extracellular Vesicles from Cytotoxic CD8+ αβ T Cells and γδ T Cells
Source: Cells. 2024 Oct 21;13(20):1745. doi: 10.3390/cells13201745 (PMC11506423; doi:10.3390/cells13201745)
Supplement: Supplementary file 1 [file cells-13-01745-s001.zip › Supplementary Fig. S2.pptx]

## Slide 1
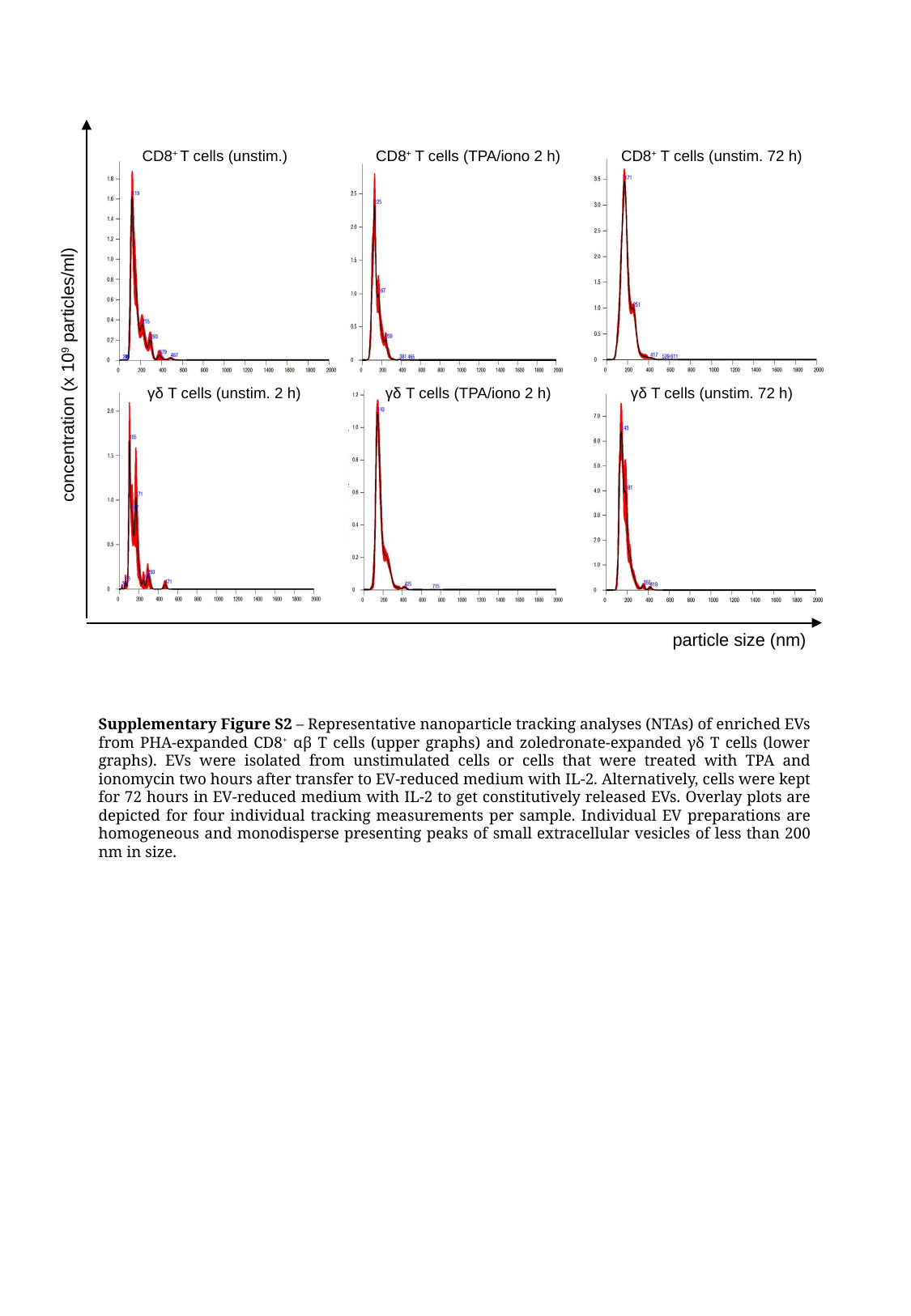

CD8+ T cells (unstim.)
CD8+ T cells (unstim. 72 h)
CD8+ T cells (TPA/iono 2 h)
concentration (x 109 particles/ml)
γδ T cells (TPA/iono 2 h)
γδ T cells (unstim. 72 h)
γδ T cells (unstim. 2 h)
particle size (nm)
Supplementary Figure S2 – Representative nanoparticle tracking analyses (NTAs) of enriched EVs from PHA-expanded CD8+ αβ T cells (upper graphs) and zoledronate-expanded γδ T cells (lower graphs). EVs were isolated from unstimulated cells or cells that were treated with TPA and ionomycin two hours after transfer to EV-reduced medium with IL-2. Alternatively, cells were kept for 72 hours in EV-reduced medium with IL-2 to get constitutively released EVs. Overlay plots are depicted for four individual tracking measurements per sample. Individual EV preparations are homogeneous and monodisperse presenting peaks of small extracellular vesicles of less than 200 nm in size.
